# Effect of biomechanical constraints in the hand laterality judgment task: where does it come from?

**DOI:** 10.3389/fnhum.2012.00299

**Published:** 2012-11-01

**Authors:** Gilles Vannuscorps, Agnesa Pillon, Michael Andres

**Affiliations:** ^1^Institute of Psychological Sciences and Institute of Neuroscience, Université catholique de LouvainLouvain-la-Neuve, Belgium; ^2^Fonds National de la Recherche ScientifiqueBrussels, Belgium; ^3^Faculty of Psychology, Department of Experimental Psychology, Ghent UniversityGhent, Belgium

**Keywords:** motor imagery, motor simulation, biomechanical knowledge, aplasia, body part perception

## Abstract

Several studies have reported that, when subjects have to judge the laterality of rotated hand drawings, their judgment is automatically influenced by the biomechanical constraints of the upper limbs. The prominent account for this effect is that, in order to perform the task, subjects mentally rotate their upper limbs toward the position of the displayed stimulus in a way that is consistent with the biomechanical constraints underlying the actual movement. However, the effect of such biomechanical constraints was also found in the responses of motor-impaired individuals performing the hand laterality judgment (HLJ) task, which seems at odds with the “motor imagery” account for this effect. In this study, we further explored the source of the biomechanical constraint effect by assessing the ability of an individual (DC) with a congenital absence of upper limbs to judge the laterality of rotated hand or foot drawings. We found that DC was as accurate and fast as control participants in judging the laterality of both hand and foot drawings, without any disadvantage for hands when compared to feet. Furthermore, DC's response latencies (RLs) for hand drawings were influenced by the biomechanical constraints of hand movements in the same way as control participants' RLs. These results suggest that the effect of biomechanical constraints in the HLJ task is not strictly dependent on “motor imagery” and can arise from the visual processing of body parts being sensitive to such constraints.

## Introduction

**Figure 1:**
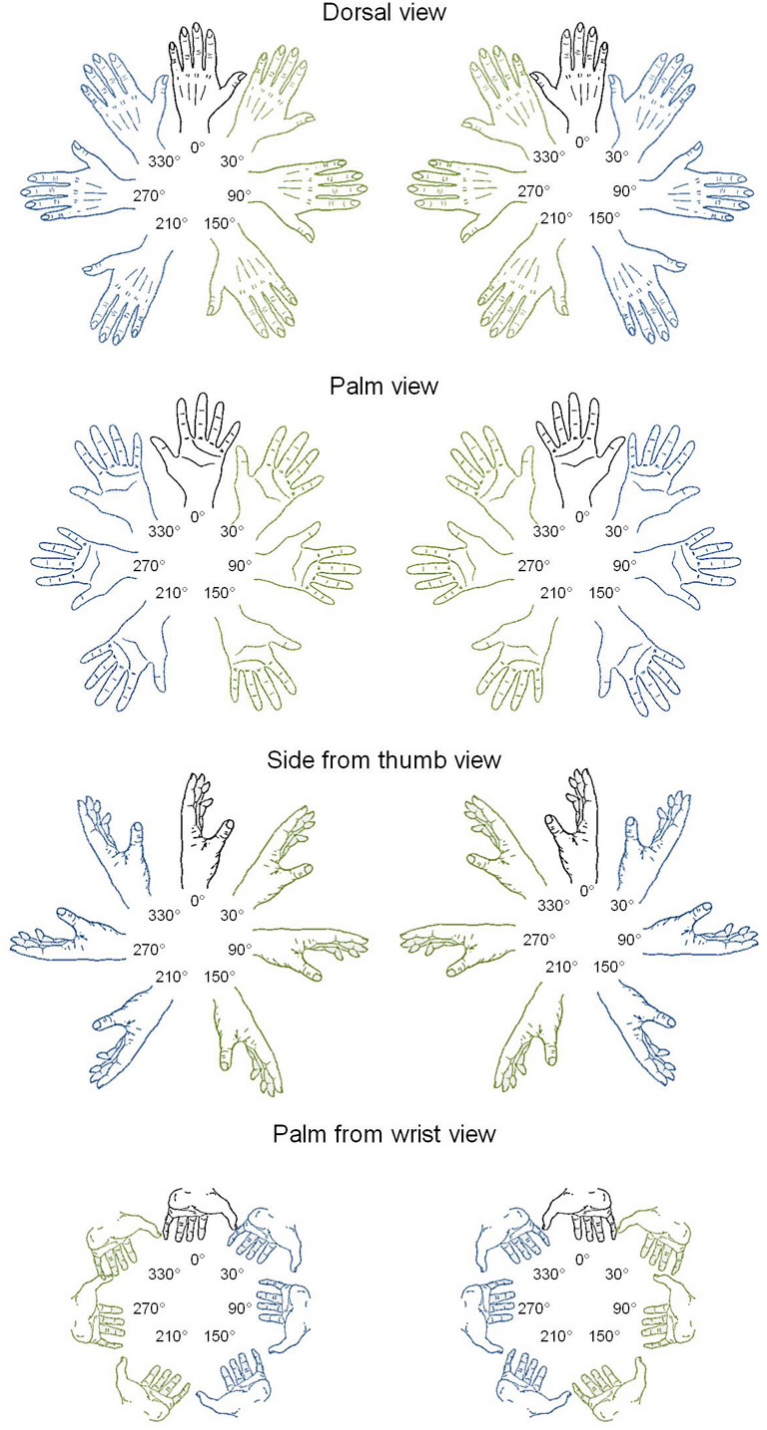
**Line drawings of left and right hands in four postures and seven rotation angles.** In blue: lateral orientations; in green: medial orientations.

In the past 20 years, an increased interest has been devoted to the study of motor imagery. Most research has been achieved by the means of the hand laterality judgment (HLJ) task (Cooper and Shepard, 1975; Sekiyama, 1982; Parsons, 1987, 1994). In this task, participants are asked to decide whether drawings of different hand postures rotated with different angles from the upright view depict a left or a right limb (see Figure 1). If a hand drawing were processed like any other visual object, response latencies (RLs) should increase as a function of the angular disparity between the hand drawing and its upright view, as evidenced for other 2D or 3D stimuli (Cooper and Shepard, 1975). Instead, it has been shown that the impact of the angular disparity on the speed of HLJs was strongly modulated by the biomechanical limits that constrain the movement of the hand toward the displayed position (Sekiyama, 1982; Parsons, 1987). This observation was taken as evidence that, to solve the task, participants internally simulate a movement of their own hand and, moreover, that mental imagery of human movements—i.e., motor imagery—relies on the same representations and processes as those involved in action planning and/or control (Parsons, 1987, 1994; Jeannerod and Frak, 1999; Kosslyn et al., 2001; Nico et al., 2004; Wraga et al., 2005; de Lange et al., 2006, 2008; Fiorio et al., 2006; Helmich et al., 2007; Munzert et al., 2009). Furthermore, neuroimaging studies showed that HLJs induced increased activity in a parieto-frontal network known for its contribution to the planning and execution of hand movements (Kosslyn et al., 1998; Parsons et al., 1998; de Lange et al., 2006). On the basis of this behavioral and neural evidence, the HLJ task was considered as a privileged tool to read out the unconscious and normally covert process of motor planning (Jeannerod, 1994; Fiorio et al., 2006; de Lange et al., 2008; Munzert et al., 2009). In this paper, we report evidence that calls for a re-examination of the prominent “motor imagery” account of the effect of biomechanical constraints in the HLJ task.

The effect of biomechanical constraints on participants' judgments in the HLJ task is characterized by three features unveiled by the analysis of the RL pattern (Parsons, 1987; Funk and Brugger, 2008; Pelgrims et al., 2009). First, the RLs for the hand drawings depicted in the various rotation angles (from 0° to 360° in a clockwise direction) from the upright display (0°) are modulated by both the specific posture of the depicted hand (e.g., view of the hand side from the thumb vs. view of the palm from the wrist) and its laterality (right vs. left hand). This three-way interaction reflects the different biomechanical constraints that limit the amplitude of the rotation angle of each hand in a given posture. Thus, for instance, the recognition of a side view takes more time for right than left hands if the drawing is rotated at 150° clockwise given that such an angle corresponds to the outcome of a possible left hand movement but it is almost impossible to achieve with the right hand in this position. Likewise, the palm of a right hand viewed from the wrist, with the thumb pointing down toward the right side of the screen (i.e., between 210° and 270° clockwise) is recognized more slowly than a left hand displayed in the same orientation, in line with the fact that it is much more difficult to reach this orientation with the right than with the left hand in this posture. The second feature indexing the effect of biomechanical constraints on participants' responses is the overall chronometric advantage for judging the laterality of hands oriented in medial positions (stimuli rotated toward the mid-sagittal plane) when compared to lateral positions (stimuli rotated away of the mid-sagittal plane). This effect, called the “Medial Over Lateral Advantage” (MOLA) effect, reflects the impact of the biomechanical constraints of hand movements that make it easier to move one's hand toward medial than lateral directions. The third feature is the significant correlation between the RL to a given stimulus and its degree of awkwardness that is, how difficult participants rated it to actually place their own hand in the displayed position (Parsons, 1987).

There is, however, evidence showing that the effect of biomechanical constraints in the HLJ task can be observed even in the condition of impaired motor planning or execution processes. After transient disruption of motor-related areas with transcranial magnetic stimulation (TMS), the performance in the HLJ task was either normal (Sauner et al., 2006) or characterized by a small RL increase that nevertheless did not hamper the effect of biomechanical constraints (Ganis et al., 2000; Pelgrims et al., 2010). Furthermore, studies of patients suffering from motor disorders that prevent normal execution of hand movements such as congenital hemiparesis (Steenbergen et al., 2007), Parkinson's disease (Helmich et al., 2007), dystonia (Fiorio et al., 2006), conversion paralysis (de Lange et al., 2008), amputation of an upper limb (Nico et al., 2004), or chronic arm pain (Schwoebel et al., 2001) showed that these conditions delayed HLJs but, again, without affecting the effect of biomechanical constraints. Finally, a study using the HLJ task with two individuals suffering from bilateral upper limb aplasia reported an effect of biomechanical constraints on RLs in at least one individual (Funk and Brugger, 2008).

Evidence for an effect of biomechanical constraints in the HLJ task despite impaired motor planning or execution raises the possibility that the effect does not arise from motor but instead from visual processes that ensure the perception of human body parts. Several authors indeed suggested that, when representing the human body in whole or in parts, the visual system encodes information about the biomechanical constraints of body part movements, information that in turn constrains whole body or body part perception (Marr and Vaina, 1982; Kourtzi and Shiffrar, 1999). Evidence advanced in support of this view includes findings from the apparent motion paradigm. When an object is displayed sequentially in two different positions, it elicits in participants the perception of an apparent motion along the shortest pathway (Kolers and Pomerantz, 1971). However, the apparent motion induced by two hand postures presented sequentially can follow a longer pathway when the shortest one is not biomechanically possible (Shiffrar and Freyd, 1990, 1993). According to some authors (Shiffrar and Freyd, 1993; Chatterjee et al., 1996), this effect indicates that implicit perceptual knowledge of how the body moves impacts how body parts are perceived (but see Stevens et al., 2000, for an alternative account in terms of motor simulation).

However, before considering the perceptual hypothesis as an alternative account for the effect of biomechanical constraints in the HLJ task, a number of ambiguities that are present in the aforementioned studies of motor-impaired or aplasic individuals must be addressed. First, one cannot rule out that the motor-impaired patients in whom the effect of biomechanical constraints was found actually suffered from a deficit affecting processes that take place *after* the stage where biomechanical constraints influence motor planning and/or execution. Indeed, motor execution was not totally abolished in these patients. At first sight, the effect found in a bilateral aplasic individual (Funk and Brugger, 2008) should not present this ambiguity since, in such condition, none of the processes involved in the planning and execution of hand movements is functional. Nevertheless, this individual (AZ) reported vivid phantom sensations of her missing body parts that include phantom movements corresponding to those of normal upper limbs (Funk et al., 2005). Thus, one cannot rule out the existence of limb representations in AZ, which could explain her ability to carry out motor imagery for congenitally absent limbs (Brugger et al., 2000; Funk and Brugger, 2008). Furthermore, and this is also true for the studies with motor-impaired patients, the issue of whether mere visual familiarity with the various hand positions could have contributed to the observed pattern of RLs in the HLJ task was not addressed. What looks like an effect of awkwardness might in fact be an artifact of the differential occurrences of the various hand positions—awkward hand positions are also likely to be less often seen than easy ones. Within this visual familiarity account, the RLs in the HLJ task would mainly depend on how often a given hand position has been seen in everyday settings, with the more frequent hand positions being recognized faster as a right or a left hand than the less frequent ones.

In this study, we sought further evidence for the presence of the effect of biomechanical constraints in the HLJ task in the context of a motor disability to plan and execute hand movements. We presented the HLJ task to DC, a man born without upper limbs, as well as to 7 normally limbed control participants, and analyzed their pattern of RLs vis-a-vis the main features indexing the effect of biomechanical constraints. Because of the congenital disability of DC, who has also never experienced any phantom limb sensation, the present study overcomes the difficulties raised by previous studies with motor-impaired patients or aplasic individuals. In the case of DC, no hand motor planning or execution ability of any kind could be invoked to explain, if any, an effect of biomechanical constraints in the HLJ task. Furthermore, we examined the effect of biomechanical constraints on the RLs of DC and control participants by taking into account the potential effect of the rated visual familiarity of hand positions.

## Materials and methods

### Participants

DC is a 51 year-old man with a Master's Degree in Psychology. He presents a congenital bilateral upper limb aplasia (right side: two fingers attached to a foreshortened humerus; left side: completely aplasic) due to *in utero* thalidomide exposure. He had no experience of prosthesis or phantom limb sensations. His performance was compared with that of 7 right-handed, normally limbed control participants matched in gender, age (mean age = 53.5), and educational level. All participants had a normal or corrected to normal vision and no history of psychiatric or neurological disorder. The study was approved by the biomedical ethic committee of the *Cliniques universitaires Saint-Luc* (Brussels) and all participants gave written informed consent prior to the study.

### Tasks and stimuli

Participants were presented with drawings of a hand outlined in black on a white background and asked to decide as fast as possible whether the drawing corresponded to a right or a left hand.

Stimuli were left or right hands, presented according to four different postures (dorsal view, palm view, side from thumb view, and palm from wrist view) and at 7 different rotation angles (upright 0° and 30°, 90°, 150°, 210°, 270°, and 330° in a clockwise direction, from Parsons, 1987; see Figure 1). The total number of different stimuli was thus of 56 hands (2 laterality × 4 postures × 7 angles). Ratings of motor awkwardness for each hand drawing were extracted from a previous study (Parsons, 1987), in which judges were asked to position their own hand at the orientation of each stimulus and, afterwards, to estimate the awkwardness of the reached position on a 5-point scale (1 = easy to place the appropriate limb into the orientation of the stimulus and 5 = difficult to place the appropriate limb into the orientation of the stimulus). Ratings of visual familiarity were collected in 25 students of the *Université catholique de Louvain* (8 males) who were asked to rate how often they saw a hand in each posture and angle of rotation in everyday life (1 = very unfamiliar and 5 = very familiar). DC's personal visual familiarity was collected separately following the same procedure; it was strongly correlated to the students' ratings [*r*_(56)_ = 0.53; *p* < 0.001]. As a control, we also tested foot laterality judgments using stimuli that were found to evoke implicit activation of the biomechanical constraints of foot movements in seminal studies of mental imagery (Parsons, 1987). Fifty-six drawings of left and right feet were presented at the same angle of rotation as hand drawings, according to four similar postures (sole view, top view, view from inside, and sole from heel view).

Participants seated in front of a computer screen located at a distance of about 60 cm; their feet were lying at rest on the ground and the hands of control participants were placed palms down on their knees without visual feedback.

During the experiment, participants performed 5 blocks of 56 trials with hand drawings and then 5 blocks of 56 trials with foot drawings. In each block, all postures and rotation angles were mixed in a different pseudo-randomized order. The first blocks of hand and foot laterality judgments included a familiarization with the four postures and 10 practice trials. Within each block, each trial started with the presentation of a central cross for 200 ms followed by a hand or foot drawing displayed until a response was recorded. Trials were separated by a blank screen of random duration between 500 ms to 1000 ms.

The experiment was controlled with the E-Prime software (Psychological Software, 2002, Pittsburgh, PA). Stimuli were presented on a 15.4 inch laptop screen set at 1024 × 768 pixels and subtended 5° of visual angle. During the testing, participants were asked to produce a verbal response (“right” or “left”). The RLs corresponded to the post-stimulus onset latency of the subject's vocalization, whose amplitude was electrically compared to a trip level voltage using a voice key controlled by E-prime. Malfunctioning of the voice key and response accuracy were monitored on-line by the experimenter.

## Results

Voice key failures (0% and 0.6% of the data in DC and controls, respectively), trials with RLs deviating more than 2 standard deviations from the mean RL within each participant (5.14% and 4.43% of the data in DC and controls, respectively), and trials with errors were discarded from RL analyses.

### General analysis of DC's and controls' performance

First of all, we performed Crawford and Howell's (1998) modified *t*-tests to test whether DC's performance (accuracy and speed) in hand and foot laterality judgments was impaired in comparison to the control group's performance. In hand judgments, no significant difference was observed between the performance of DC (correct responses: 97%; mean RL: 1237 ms) and control participants [mean % correct responses ± SD: 91% ± 4%, modified *t*_(6)_ = 1.56, *p* > 0.1; mean RL ± SD: 1277 ms ± 345 ms, modified *t*_(6)_ = −0.11, *p* > 0.9]. The analysis of foot judgments showed a similar pattern, with a non-significant difference in accuracy or RLs between DC (correct responses: 97%; mean RL: 1512 ms) and controls [mean % of correct responses ± SD: 90% ± 6%, modified *t*_(6)_ = 1.13, *p* > 0.3; mean RL ± SD: 1446 ms ± 386 ms, modified *t*_(6)_ = 16, *p* > 0.8]. Second, Crawford and Garthwaite's (2005) Revised Standardized Difference Test (RSDT) was applied to test whether the difference between hand and foot laterality judgments in DC deviated from the difference observed between hand and foot judgments in the control group. The results showed that DC's difference in performance between hand and foot drawings was not significantly different from that found in the control participants, either in accuracy [RSDT: *t*_(6)_ = 0.31, *p* > 0.7] or speed [RSDT: *t*_(6)_ = 0.4, *p* > 0.7]. Third, independent samples *t*-tests were performed in order to compare the RLs across posture, laterality, and angle of rotation for foot and hand judgments in DC and controls, respectively. The results indicated that both DC [*t*_(67.76)_ = −2.99, *p* < 0.01] and control participants [*t*_(84.1)_ = −4.29, *p* < 0.01] were significantly faster for hand than for foot judgments.

### Effect of biomechanical constraints on DC's and controls' RLs

Having shown that the performance of DC in HLJs was within the normal range, we looked for the presence of the three behavioral features that were classically reported as evidence for an effect of biomechanical constraints in the HLJ task.

First, we investigated the presence of a three-way interaction between laterality, angle, and posture in the chronometric data gathered for all participants. To do so, the RLs of control participants were entered in a repeated measure analysis of variance (ANOVA) with subject as the random factor and laterality (left vs. right), posture (dorsal view vs. palm view vs. palm from the wrist view vs. side from the thumb view), and angle with respect to the upright view (0°–330°, in a clockwise direction) as within-subject factors. The data of control participants required a log transformation to satisfy the ANOVA's homoscedasticity and normality assumptions. In order to explore the effects of biomechanical constraints in DC's judgments, we performed an ANOVA with item as the random factor and laterality (left vs. right), posture (dorsal view vs. palm view vs. palm from the wrist view vs. side from the thumb view), and angle with respect to the upright view (0°–330°, in a clockwise direction) as between-item factors. For the analysis of DC's performance, the data were inverse transformed to fulfill the criteria of homoscedasticity and normality. The results replicated the significant three-way interaction between laterality, angle, and posture, not only in control participants [*F*_(18, 108)_ = 3.77, *p* < 0.001] but also in DC [*F*_(18, 202)_ = 2.69, *p* < 0.001]. This three-way interaction showed that the effect of the angle of rotation on RLs was not symmetric for left and right hands, with the angle associated to the maximal increase varying as a function of hand posture.

**Figure 2:**
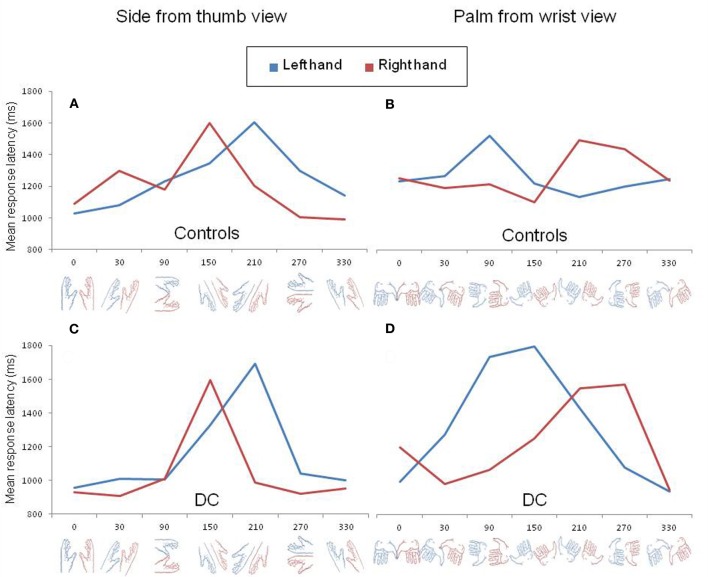
**Mean response latency for left (blue) and right (red) hand drawings rotated clockwise by steps of 60° viewed from the side (A,C) and from the wrist (B,D).** The pattern of response latency in control participants and DC are shown in the upper (**A,B**) and lower (**C,D**) panels, respectively.

In order to further exemplify the effect of biomechanical constraints, we decomposed the three-way interaction as a function of hand posture. In control participants, the log transformed data were analyzed separately for each hand posture using repeated measure ANOVAs with subject as a random factor and laterality and angle as within-subject factors. The inverse transformed data of DC were entered in similar ANOVAs with item as a random factor and laterality and angle as between-item factors. In control participants, a significant laterality by angle interaction was found for the palm from the wrist view [*F*_(6, 36)_ = 4.32, *p* < 0.01] and for the palm view [*F*_(6, 36)_ = 7.68, *p* < 0.001]. A near significant interaction effect was observed for the side from the thumb view [*F*_(6, 36)_ = 2.15, *p* = 0.07] but, for the dorsal view, no significant interaction was found [*F*_(6, 36)_ = 1.38, *p* > 0.2]. The performance of DC also revealed a significant laterality by angle interaction for the palm from wrist view [*F*_(6, 51)_ = 3.98, *p* < 0.01] as well as for the side from the thumb view [*F*_(6, 56)_ = 2.5, *p* < 0.05], but not for the palm [*F*_(6, 41)_ = 1.49, *p* > 0.2] or dorsal view [*F*_(6, 54)_ < 1]. In Figure 2, we represented the pattern of RLs of control participants (upper panel) and DC (lower panel) for the two postures that showed a significant laterality by angle interaction in DC after decomposition of the three-way interaction. The Figure clearly illustrates that the angle of rotation had a different effect on RLs depending on hand laterality. In both controls (Figure 2A) and DC (Figure 2C), the side view of a left hand led to maximal RLs at angle 210°, whereas the slowest responses for the side view of a right hand were observed at angle 150°. In contrast, the palm from the wrist views showing the longest RLs ranged from angles 90° to 150° for the left hand and from angles 210° to 270° for the right hand (Figures 2B,D). The comparison of Figures 2B and 2D showed that maximal RLs for the palm from the wrist views were not observed exactly at the same angles in controls and DC, with a RL curve skewed to angle 150° for DC. Except for this slight difference, the angles associated to maximal RLs in DC and controls reflect the most difficult positions to reach while adopting the displayed posture with one's left or right hand (see the awkwardness estimates provided by Parsons, 1987).

DC did not show the expected pattern of asymmetric RL curves for the left and right hands viewed from the dorsal or from the palm view. However, the absence of laterality by angle interaction for these two postures was already pointed out in previous experiments with normally limbed participants (Parsons, 1987; see for discussion ter Horst et al., 2010). Likewise, in our control participants, the effect of angular disparity was not modulated by hand laterality for dorsal views and a look at the individual data for the palm views revealed that the expected interaction was observed in only 3 out of 7 participants.

**Figure 3:**
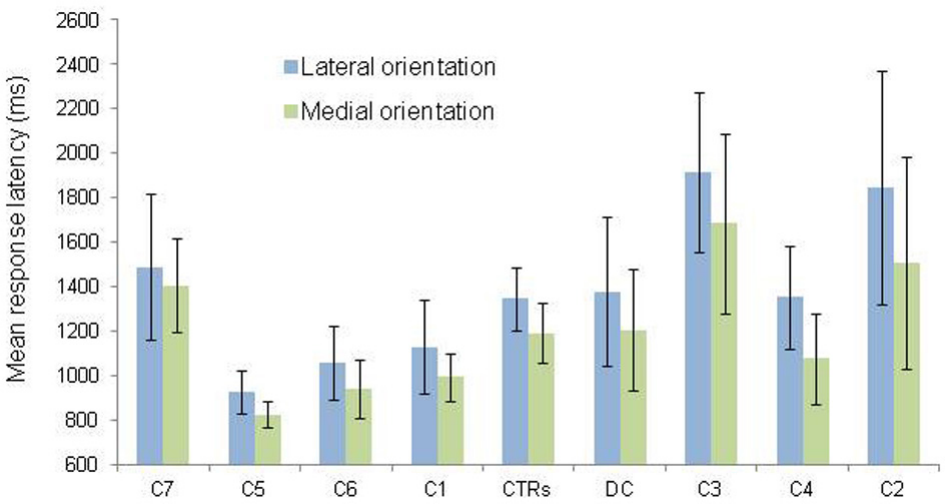
**Mean response latency and standard deviation (vertical bars) are shown as a function of hand orientation (lateral vs. medial).** The data for the lateral and medial orientation were obtained by pooling the data from all postures and rotation angles corresponding to the lateral (blue) and medial (green) positions depicted in Figure 1. Individual data (C = Control; CTRs = mean of controls) are aligned in ascending order on the X axis as a function of the size of the medial over lateral advantage.

Second, in order to test for the presence of the MOLA effect, we recoded the trials according to the “medial” or “lateral” orientation of the displayed hand posture with respect to the body mid-sagittal plane (cf. Figure 1: medial orientations are displayed in green and lateral orientations in blue). In that way, the analyses were performed on the mean RLs calculated for each 24 medial and each 24 lateral hand displays (2 hands × 3 angles × 4 postures). Figure 3 shows the mean RLs associated to medial and lateral orientations in DC and every control participant. Independent samples *t*-tests revealed that both control participants [medial: 1191 ms ± 134 ms; lateral: 1345 ms ± 139 ms; *t*_(46)_ = 3.89, *p* < 0.001] and DC [medial: 1205 ms ± 274 ms; lateral 1378 ms ± 335; *t*_(46)_ = 1.95, *p* = 0.057] responded faster to hand drawings in medial than in lateral orientation, which mimicked the effect of biomechanical constraints on actual hand movements. Furthermore, RSDT indicated that the MOLA effect did not significantly differ in size between DC and controls [RSDT: *t*_(6)_ = 0.13, *p* > 0.9].

**Figure 4:**
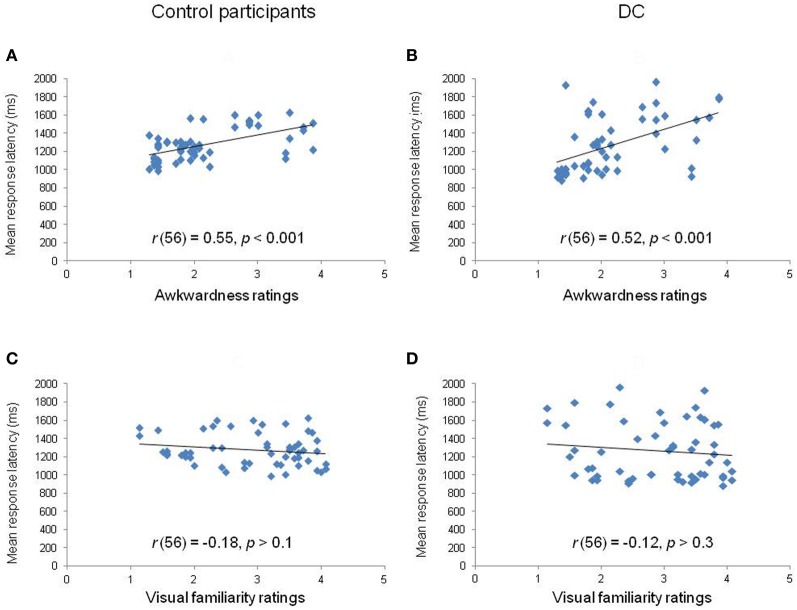
**Correlation plots between the mean response latency of controls (left panels) and DC (right panels) for each hand stimulus in relation to rated awkwardness (A,B) and visual familiarity (C,D)**.

Third, we calculated the correlation between the mean RL obtained for each of the 56 items and the estimates of motor awkwardness collected independently for the same items (Parsons, 1987). The correlation plots are displayed in Figures 4A,B. The analyses showed that the RLs of both controls [*r*_(56)_ = 0.55, *p* < 0.001] and DC [*r*_(56)_ = 0.52, *p* < 0.001] were significantly correlated to the motor awkwardness of the hand drawing. A final set of analyses was performed in order to partial out the respective influence of motor awkwardness and visual familiarity on RLs (see Figures 4C,D). We conducted stepwise regression and partial correlation analyses between RLs and, respectively, ratings of motor awkwardness and visual familiarity. The regression analysis performed in control participants [*F*_(1, 54)_ = 23.94, *p* < 0.001, *R*^2^ = 0.31] showed that the motor awkwardness associated to a given hand drawing was the best predictor of the observed RLs (β = 0.55, *t* = 4.89, *p* < 0.001). The contribution of visual familiarity was not significant (β = −0.04, *t* = −0.35, *p* > 0.7). Likewise, the regression of DC's RLs [*F*_(1, 54)_ = 19.87, *p* < 0.001, *R*^2^ = 0.27] revealed a significant effect of motor awkwardness (β = 0.52, *t* = 4.46, *p* < 0.001) in the context of a non-significant contribution of visual familiarity (β = 0.01, *t* = 0.11, *p* > 0.9). Similar results were observed when DC's own ratings of visual familiarity were taken into account in the regression equation [*F*_(1, 54)_ = 19.87, *p* < 0.001, *R*^2^ = 0.27]. Furthermore, partial correlations showed that the correlation between RLs and awkwardness estimates remained significant in controls [*r*_(53)_ = 0.54, *p* < 0.001] and DC [*r*_(53)_ = 0.51, *p* < 0.001] after controlling for the part of variance explained by the effect of visual familiarity.

### Summary of results

The goal of this study was to test whether the effect of biomechanical constraints in the HLJ task is strictly dependent on hand motor planning and execution abilities. To do so, we compared the performance of DC, a bilateral aplasic individual, with the performance of control participants in the HLJ task. First, we looked at the overall performance and found that DC was as fast and accurate as the control participants. Second, we found that (1) DC's RLs were influenced by the laterality, the angle, and the posture of hand stimuli, in a way that mirrors the biomechanical limits imposed by each posture on left and right hand movements; (2) DC's RLs showed a chronometric advantage of medial over lateral hand orientations, reflecting the difference of movement amplitude allowed by these two orientations; and (3) DC's RLs were strongly predicted by the motor awkwardness of the stimuli but not by their visual familiarity. To sum up, DC showed the three behavioral features that were classically reported as evidence for an effect of biomechanical constraints in the HLJ task. These effects were qualitatively and quantitatively comparable to the effects observed in control participants and they cannot be explained by the differential occurrences of the various hand positions in everyday life.

## Discussion

The effect of biomechanical constraints in the HLJ task is commonly assumed to reflect a process of motor simulation anchored in the same processes and representations as those involved in actual action planning and execution (Parsons, 1987, 1994; Jeannerod and Frak, 1999; Kosslyn et al., 2001; Nico et al., 2004; Wraga et al., 2005; de Lange et al., 2006, 2008; Fiorio et al., 2006; Helmich et al., 2007; Munzert et al., 2009). In this paper, we found that a person born without upper limbs was as accurate and fast as a group of control participants in performing the HLJ task and that his RLs in this task were significantly influenced by the biomechanical constraints of upper limb movements, just like the RLs of normally limbed participants. These findings show that the effect of biomechanical constraints in the HLJ task is not strictly dependent on representations and processes involved in the planning and execution of hand movements. Given his total lack of motor experience with upper limb movements, and also of phantom limb experience, DC is not endowed with such motor representations and processes and could therefore not rely on them to perform the task.

Two kinds of accounts for the effect of biomechanical constraints in DC can be dismissed. First, this effect is not an artifact of the visual familiarity of the various hand positions. We found that DC's RLs were better predicted by the degree of motor awkwardness associated to each hand drawing than by their visual familiarity. Furthermore, the correlation between his RLs and motor awkwardness estimates remained significant even after removing the influence of visual familiarity on the data, which indicated that the influence of motor awkwardness and visual familiarity does not fully overlap in HLJs. Second, our data allow us to rule out that the effect is due to DC performing the HLJ task by mentally rotating the representation of his feet. Seminal studies in fact showed that the motor awkwardness estimates associated to certain foot positions correlate with those gathered for the homologue hand positions (see Tables 3 and 4 in Parsons, 1987). However, DC was 275 ms faster for hand than foot judgments. This advantage of hand over foot responses even reached an average of 583 ms for the side views that showed the typical effect of biomechanical constraints^1^.

Our findings thus provide strong evidence for the presence of an effect of biomechanical constraints in the HLJ task in a condition that totally prevents the planning and execution of hand movements. Uncovering the source of this effect in such condition was beyond the scope of this study and we have no direct evidence that speaks to this issue. Nevertheless, the finding that knowledge of biomechanical constraints was implicitly and automatically recruited in DC's HLJs is consistent with the view that such knowledge is an intrinsic component of body part visual perception processes. These perceptual processes would provide us with a representation of the human body that takes into account information about the range of movement allowed by the different body parts (Marr and Vaina, 1982; Shiffrar and Freyd, 1990, 1993; Kourtzi and Shiffrar, 1999). Such information might have an adaptive value for humans because it facilitates the anticipation of the outcome of movements performed by others (Kourtzi and Shiffrar, 1999). The type of body representation we propose to explain the results of DC should not be confused with the “body schema” because this representation refers specifically to one's own body (Corradi-Dell'Acqua and Tessari, 2010; de Vignemont, 2010). It is also different from the “body structural description” (i.e., a visuospatial representation of body parts) and the “body image” (i.e., a conceptual representation of the body) because none of these representations include knowledge of the biomechanical constraints of the body (Sirigu et al., 1991; Schwoebel and Coslett, 2005).

The role of visual processes in the effect of biomechanical constraints in the perception of body parts was already emphasized by Brugger and colleagues in order to explain the influence of such constraints on HLJs (Brugger et al., 2000; Funk and Brugger, 2008) and apparent motion perception (Funk et al., 2005) in an bilateral aplasic individual, AZ, who experienced phantom limb movement sensations. It should be noted, however, that this proposal deviates from the idea that biomechanical knowledge is an intrinsic component of visual perception. In Brugger and colleagues' proposal, visual experience is assumed to activate pre-existing limb representations common to both action observation and execution, thereby allowing AZ to engage in a process of motor imagery in the HLJ task. Our finding that biomechanical constraints also affect the performance of a bilateral aplasic individual *without* phantom limb sensations in the HLJ task makes it unnecessary to assume that the role of visual experience is mediated by processes involved in action planning or execution.

In their study, Funk and Brugger (2008) also presented the HLJ task to a bilateral aplasic individual, CL, who did *not* experience phantom limb sensations, like DC, but, contrary to the results we reported here, they found no evidence for an effect of biomechanical constraints in CL's response pattern. These discrepant results in the HLJ task deserve some methodological considerations. While performing the task, CL was influenced by the rotation angle but not by the biomechanical constraints, as evidenced by an absence of interaction between hand laterality and rotation angle. However, CL was tested with a short version of the HLJ task including only two postures (palm vs. back) and four rotation angles (from 0° to 270° by steps of 90°). Previous research in healthy participants showed that minor modifications in the stimulus set, such as the reduction of the number of rotation axes, can suppress the effect of biomechanical constraints (ter Horst et al., 2010). Furthermore, laterality judgment for hands viewed from the back and the palm are less regularly associated with an effect of the biomechanical constraints, even in normally limbed participants (e.g., Parsons, 1987; this study). It is therefore unclear whether the effect of biomechanical constraints in CL's performance was not observed because he had no motor experience or because the stimulus set allowed him to base his responses on strategic processes that do not make use of biomechanical knowledge. This observation underlines the need to assess the influence of biomechanical knowledge on HLJs with an extended set of hand drawings.

Finally, our findings call for re-examining the motor imagery account for the effect of biomechanical constraints in normally limbed individuals performing HLJs. Obviously, the effect observed in DC and controls could be driven by distinct kinds of representations and processes, i.e., visual vs. motor processes, both being sensitive to the biomechanics of the human body. Nevertheless, we find it unlikely that an implicit and automatic access to biomechanics, such as the one revealed in DC's laterality judgments, would be totally suspended when normal subjects perform the task. Our findings thus encourage further empirical studies to entertain the hypothesis that visual in addition to motor processes, or even visual processes alone, contribute to the effect of biomechanical constraints in the HLJ task. In the meanwhile, the HLJ task should not be considered anymore as an unambiguous window on the covert stages of motor control and motor planning in normal or motor-impaired individuals.

### Conflict of interest statement

The authors declare that the research was conducted in the absence of any commercial or financial relationships that could be construed as a potential conflict of interest.

## Footnotes

^1^Several authors (e.g., Tomasino et al., 2003; Steenbergen et al., 2007; Daprati et al., 2010; ter Horst et al., 2010) argued that, when motor simulation is not possible (e.g., aplasia or severe motor deficit), alternative strategies may be used to perform the HLJ task. These strategies imply, for instance, mental rotation of the visual stimulus (Tomasino et al., 2003), visual analysis based on non-motor features of the hands such as the number of visible fingers or the position of the thumb and/or pinkie (Daprati et al., 2010), or third-person visual imagery of the action “as if someone else performed the action” (Steenbergen et al., 2007). It is important to note, however, that all these visually based strategies predict no effect of biomechanical constraints on response latencies. Thus, any account for DC's pattern in the HLJ task in terms of such alternative strategies would be inappropriate.
